# A Near-Infrared Fluorescent Probe for Specific Imaging of Lymph Node Metastases in Ovarian Cancer via Active Targeting of the Gonadotropin-Releasing Hormone Receptor

**DOI:** 10.3390/biom15060868

**Published:** 2025-06-14

**Authors:** Qiyu Liu, Jiaan Sun, Xiaobo Zhou, Mingxing Zhang, Tao Pu, Xiaolan Gao, Meng Zhang, Congjian Xu, Xiaoyan Zhang

**Affiliations:** 1 Obstetrics and Gynecology Hospital of Fudan University, Shanghai 200011, China; 2 Department of Chemistry, Fudan University, Shanghai 200438, China; 3 Shanghai Key Laboratory of Female Reproductive Endocrine Related Diseases, Shanghai 200011, China; 4 Department of Obstetrics and Gynecology of Shanghai Medical School, Fudan University, Shanghai 200032, China

**Keywords:** intraoperative imaging agents, tumor targeting, ovarian cancer, gonadotropin-releasing hormone, lymph node metastasis

## Abstract

Lymph node metastases are common in advanced ovarian cancer and are associated with poor prognosis. Accurate intraoperative identification of lymph node metastases remains a challenge in ovarian cancer surgery due to the lack of tumor-specific intraoperative imaging tools. Here, we developed a gonadotropin-releasing hormone receptor (GnRHR)-targeted near-infrared (NIR) fluorescent probe, GnRHa-PEG-Rh760, through conjugation of a GnRH analog peptide with the Rh760 fluorophore and polyethylene glycol (PEG). A non-targeted probe (PEG-Rh760) served as control. In mouse models of subcutaneous xenografts, peritoneal and lymph node metastases derived from ovarian cancer cells, GnRHa-PEG-Rh760 showed superior tumor-specific accumulation. NIR fluorescence imaging revealed strong fluorescence signals localized to primary tumors, peritoneal lesions, and metastatic lymph nodes with no off-target signals in normal lymph nodes. The spatial co-localization between the NIR fluorescence of GnRHa-PEG-Rh760 and tumor-derived bioluminescence clearly confirmed the probe’s target specificity. GnRHa-PEG-Rh760 mainly accumulated in the tumor and liver and was gradually cleared at 96 h post-injection. The retention of fluorescence signals in normal ovary tissue further validated GnRHR-mediated binding of the probe. Notably, GnRHa-PEG-Rh760 exhibited excellent biocompatibility with no observed systemic toxicity as evidenced by hematologic and histopathologic analyses. These data demonstrate the potential of GnRHa-PEG-Rh760 as an intraoperative imaging agent, providing real-time fluorescence imaging guidance to optimize surgical precision. This study highlights the value of receptor-targeted molecular imaging probes in precision cancer surgery.

## 1. Introduction

Cancer remains the common leading cause of death worldwide. Ovarian cancer is the most lethal gynecological malignancy in the United States [1]. Over 80% of patients with ovarian cancer are diagnosed at advanced stages, with a 5-year survival rate of 42% for patients at stage III and 26% for patients at stage IV [2,3]. Cytoreductive surgery for removing all visible or palpable tumors is the key component in the care of advanced ovarian cancer patients. Patients with no residual disease (R0) after surgery have the best survival outcome [4,5]. However, the R0 resection rate was only 17% in patients after primary cytoreductive surgery and 39% in patients after interval cytoreductive surgery [6].

Real-time guidance provided by intraoperative fluorescence imaging helps improve the resection rate of tumors [7,8]. Over the past two decades, researchers have attempted to develop tumor-specific fluorophores for intraoperatively detecting ovarian cancer [9]. Pafolacianine (OTL38), a fluorescent tracer that targets folate receptor alpha, is the first FDA-approved agent for fluorescence-guided ovarian cancer surgery. In a phase III study (NCT03180307), additional cancer lesions were identified in 33% of patients with known or suspected ovarian cancer who were scheduled to undergo cytoreductive surgery by pafolacianine with near-infrared (NIR) fluorescence imaging in contrast to white light assessment and palpation [10]. However, false-positive lymph nodes were also observed in phase I to III studies of pafolacianine, which was mostly due to the expression of folate receptor beta in the noncancerous lymph nodes [10,11,12,13].

Lymph node metastases are commonly observed in advanced ovarian cancer and are associated with a poor survival rate [14,15,16]. Systematic lymphadenectomy seems necessary to achieve complete tumor reduction. However, systematic lymphadenectomy does not improve overall survival in patients with optimally debulked advanced ovarian cancer [17]. In a prospectively randomized, controlled trial of lymphadenectomy in patients with primary ovarian cancer (FIGO stage IIB through IV) with macroscopically complete resection and clinically negative lymph nodes (LION trial), systematic pelvic and paraaortic lymphadenectomy did not improve overall or progression-free survival but was instead associated with a higher incidence of postoperative complications [18]. Although sentinel lymph node (SLN) mapping is effective in assessing lymph node metastasis in patients with endometrial and cervical cancer and may allow the avoidance of systematic lymphadenectomy, the benefit of SLN mapping in patients with ovarian cancer remains controversial [19,20]. The reasons probably include the anatomical location of the ovaries, complex lymphatic drainage and different lymphatic spread pathways of ovarian cancer. A multicenter, prospective, phase II trial (SELLY) evaluated the sensitivity and specificity of SLN mapping using indocyanine green (ICG) compared with systematic lymphadenectomy in detecting lymphatic metastases in early-stage ovarian cancer and reported a low detection rate without achieving the expected sensitivity [21]. Intraoperative recognition and selective resection of metastatic lymph nodes are crucial for adequate staging and adjuvant treatment but remain challenging in patients with ovarian cancer.

The overexpression of gonadotropin-releasing hormone (GnRH) receptor (GnRHR) in ovarian cancers makes it an effective theranostic target. Our previous study confirmed the overexpression of GnRHR in ovarian cancer and the binding capacity of the GnRHa peptide to ovarian cancer cells [22]. Here, we report the development and validation of a GnRHR-targeted NIR fluorescent probe, GnRHa-PEG-Rh760, as a tool for the intraoperative specific fluorescence imaging of metastatic lymph nodes in ovarian cancer. The probe was synthesized with a GnRH analog (GnRHa) peptide capable of binding to GnRHR, the Rh760 fluorophore, and polyethylene glycol (PEG). The GnRHa-PEG-Rh760 probe specifically recognized and illuminated primary tumors, peritoneal metastases and lymph node metastases of ovarian cancer from normal tissues with no detectable systemic toxicity.

## 2. Materials and Methods

### 2.1. Cell Culture

Human ovarian cancer cell lines (A2780 and ES-2) were used (archived in our laboratory). Firefly luciferase-expressing A2780 cells (A2780-luciferase) and ES-2 cells (ES-2-luciferase) were generated by transducing lentiviral particles (Obio Technology, Shanghai, China). A2780 and A2780-luciferase cells were cultured in RPMI-1640 medium supplemented with 10% fetal bovine serum (FBS). ES-2 and ES-2-luciferase cells were cultured in McCoy’s 5A medium supplemented with 10% FBS.

### 2.2. Synthesis and Characterization of GnRHa-PEG-Rh760

**Figure 1:**
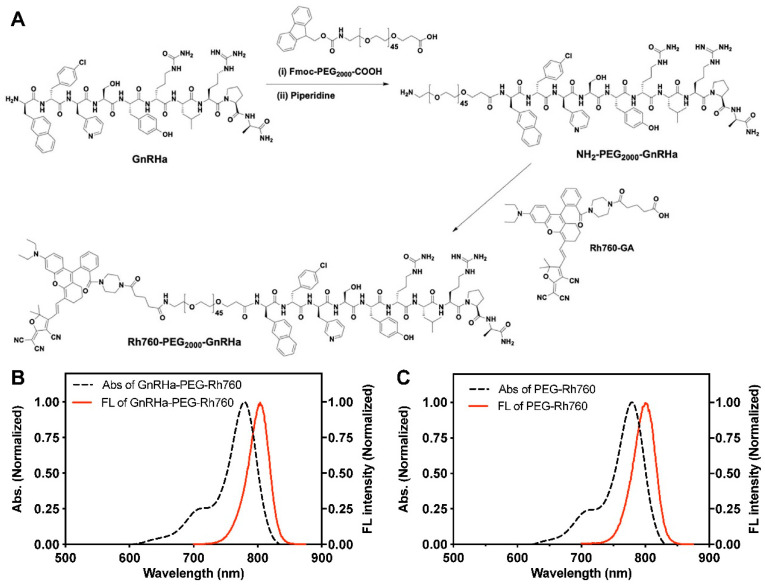
Synthesis and characterization of GnRHa-PEG-Rh760 and PEG-Rh760. (**A**) Synthetic schematic diagram of GnRHa-PEG-Rh760. UV–visible absorption spectra (Abs) and fluorescence emission spectra (Fl) of GnRHa-PEG-Rh760 (**B**) and PEG-Rh760 (**C**).

The probe GnRHa-PEG-Rh760 was synthesized as shown in the scheme in Figure 1A. The NIR fluorophore Rh760 and its derivatives Rh760-PAZ and Rh760-GA were synthesized as previously reported [23,24]. All probes were synthesized by Guoping Pharmaceutical (Hefei, China). Briefly, the sequence of the GnRHa peptide (D-2-Nal-D-4-Cl-Phe-D-3-Pal-Ser-Tyr-D-Cit-Leu-Arg-Pro-D-Ala-NH_2_) was prepared via the standard Fmoc solid-phase peptide synthesis method. After peptide chain coupling, piperidine was added, and the mixture was allowed to react for 20 min to remove Fmoc. Then, Fmoc-PEG2000-COOH and Rh760 were added in sequence and allowed to react until the resin test was colorless. The resin was washed with methanol and then shaken for 2 h with cutting fluid. The resulting reaction mixture was precipitated with ice-cold ether, yielding a blue–green solid. PEG-Rh760 without GnRHa peptide modification was synthesized as the control. The final products were evaluated by mass spectrometry (MS). The UV–visible absorption spectra were measured with a LAMBDA 750 spectrometer (PerkinElmer, Waltham, USA), and the fluorescence emission spectra were measured with a QuantaMaster 40 fluorospectrometer in DMSO solution.

### 2.3. Establishment of Animal Models

To evaluate in vivo tumor imaging, mouse models with subcutaneous xenografts, peritoneal and lymph node metastases were established separately using ovarian cancer cell lines. Five-week-old female BALB/c nude mice were used. The experiments were approved by the Animal Welfare and Ethics Committee of Department of Laboratory Animal Science of Fudan University.

Mouse models with subcutaneous xenografts: A total of 5 × 10^6^ A2780 cells were subcutaneously injected into the flanks of the mice. The tumors were allowed to grow until their diameter reached approximately 5 mm before treatment.

Mouse models with peritoneal metastasis: A total of 1 × 10^7^ A2780-luciferase cells were injected into the abdominal cavities of the mice. The tumors were allowed to grow for 2 weeks and monitored via bioluminescence imaging using the IVIS Lumina K Imaging system (PerkinElmer, Waltham, USA).

Mouse models with lymph node metastasis: A total of 2 × 10^6^ ES-2-luciferase cells were injected into the unilateral footpads of the mice. The lymph node metastases were monitored via bioluminescence imaging. Spontaneous metastases to popliteal (PO) and inguinal (IN) lymph nodes as well as occasional metastases to iliac (IL) and sciatic (SC) lymph nodes were observed after 2–3 weeks of primary tumor growth. The lymph node tissues were confirmed by histological examination and immunohistochemical staining for CK7.

### 2.4. Immunohistochemical Staining

Tumor and lymph node tissues were fixed with formalin, embedded in paraffin, and then sectioned. The dewaxed tissue sections were incubated with anti-GnRHR (Cat# ab220196, 1:100 dilution, Abcam, Cambridge, UK) or anti-CK7 (Cat# ab181598, 1:8000 dilution, Abcam, Cambridge, UK) primary antibodies. Then, the tissue sections were washed and separately incubated with the corresponding HRP-conjugated secondary antibodies (Cat# 115-035-003 and Cat# 111-035-003, 1:500 dilution, Jackson ImmunoResearch, Lancaster, USA). Finally, the tissue sections were stained with diaminobenzidine (Cat# 8059P, CST, Danvers, USA) and hematoxylin.

### 2.5. Confocal Fluorescence Microscopy

To examine the binding of probes to ovarian cancer tissues, tumors, lymph nodes and skeletal muscle were obtained from sacrificed mice and then embedded in frozen section embedding medium. The frozen tissues were sectioned, fixed and mounted. Nuclei were labeled with DAPI. Fluorescence images were captured via confocal fluorescence microscopy (TCS SP5, Leica, Wetzlar, Germany).

### 2.6. In Vivo and Ex Vivo NIR Fluorescence Imaging

The tumor-bearing mice were injected with GnRHa-PEG-Rh760 at a dose of 2.0 mg/kg body weight (n = 3). PEG-Rh760 was used as a non-targeted control probe. The probes were formulated using phosphate buffered saline (PBS) solutions with a pH of 7.4. For mice with subcutaneous xenografts and lymph node metastasis, the probe was administered via tail vein injection. For mice with peritoneal metastasis, the probe was injected intraperitoneally into the lower right abdominal region. In vivo NIR fluorescence imaging (Ex/Em = 740 nm/790 nm) was performed at different time points post-injection via the IVIS Lumina K Imaging system (PerkinElmer, Waltham, USA). Moreover, tumors, lymph nodes, and major organs were immediately dissected from sacrificed mice and imaged under NIR fluorescence. The fluorescence signals were quantified as the average radiant efficiency ([p/s/cm^2^/sr]/[μW/cm^2^]) via Living Image software (version 4.5, PerkinElmer, Waltham, USA).

### 2.7. In Vivo and Ex Vivo Bioluminescence Imaging

To verify the specificity of the NIR fluorescence imaging of GnRHa-PEG-Rh760, bioluminescence imaging of A2780-luciferase and ES-2-luciferase tumor-bearing mice was performed via the IVIS Lumina K Imaging system (PerkinElmer, Waltham, USA). The mice were injected with 150 mg/kg body weight D-luciferin (YEASEN Biotech, Shanghai, China) and imaged at 15 min post-injection. Moreover, tumors, lymph nodes, and major organs were immediately dissected from sacrificed mice and imaged. Bioluminescence signals were quantified as the total flux (p/s) via Living Image software (version 4.5, PerkinElmer, Waltham, USA).

### 2.8. In Vivo Safety Evaluation

The mice were injected with GnRHa-PEG-Rh760 at a dose of 2.0 mg/kg body weight via the tail vein (n = 3). PEG-Rh760 and vehicle were used as controls. The body weights of the mice were continuously monitored. The mice were sacrificed after 6 days. Routine blood tests were conducted to assess red blood cells (RBCs), white blood cells (WBCs), and platelets (PLTs). Blood biochemistry tests were conducted to assess alanine transaminase (ALT), aspartate transaminase (AST), blood urea nitrogen (BUN), and creatinine (CREA) levels. Tissue sections from organs, including the heart, lung, liver, spleen, kidney, and muscle, were stained with hematoxylin and eosin (H&E) for histopathological analysis.

### 2.9. Statistical Analysis

All quantitative data are presented as mean ± standard deviation (SD). Two-group comparisons were performed using the unpaired Student’s *t*-test. Statistical significance was defined as *p*-value < 0.05. Statistical analyses were conducted using GraphPad Prism software (version 10.2.0).

## 3. Results

### 3.1. Synthesis and Characterization of GnRHa-PEG-Rh760 and PEG-Rh760

GnRHa-PEG-Rh760 was synthesized as shown in Figure 1A. The GnRHa peptide capable of binding to GnRHR was modified with PEG and then covalently conjugated to Rh760. PEG-Rh760 was also synthesized as a control probe. The mass spectrometry evaluation is shown in Supplementary Figure S1. The average molecular weight was 4000 Da for GnRHa-PEG-Rh760 and 2500 Da for PEG-Rh760. In DMSO solution, GnRHa-PEG-Rh760 and PEG-Rh760 presented the same spectra, with a maximum absorption peak at 780 nm and a maximum emission peak at 805 nm (Figure 1B,C). The results showed that the spectra of PEG-Rh760 were not affected by the conjugation of the GnRHa peptide.

### 3.2. Specific Accumulation of GnRHa-PEG-Rh760 in Primary Tumors

**Figure 2:**
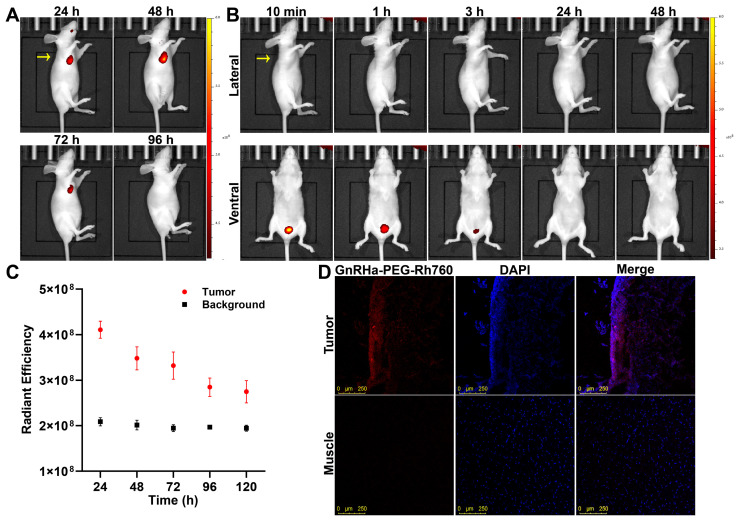
Specific accumulation of GnRHa-PEG-Rh760 in subcutaneous ovarian cancer xenografts. Mice bearing subcutaneous A2780 ovarian cancer were injected with GnRHa-PEG-Rh760 or PEG-Rh760 via the tail vein. (**A**) Representative in vivo NIR fluorescence images of GnRHa-PEG-Rh760. (**B**) Representative in vivo NIR fluorescence images of PEG-Rh760. The lateral and ventral sides of the same mouse were shown. The yellow arrow indicates the tumor location. (**C**) Quantitative ex vivo fluorescence intensity analysis of tumors relative to skeletal muscle for GnRHa-PEG-Rh760 (n = 3). (**D**) Representative confocal fluorescence microscopy images of frozen sections from the tumor and skeletal muscle of the mice injected with GnRHa-PEG-Rh760. Bar: 250 μm.

First, we evaluated the accumulation of GnRHa-PEG-Rh760 in primary tumors using a mouse model bearing subcutaneous A2780 (GnRHR-positive) ovarian cancer. Representative in vivo fluorescence images at different time points are presented in Figure 2A,B. High fluorescence intensity was observed in the subcutaneous tumors of the GnRHa-PEG-Rh760 group. By contrast, in the PEG-Rh760 group, no fluorescence was detected in the tumors even at 48 h post-injection; only the bladder exhibited fluorescence at 10 min, 1 h, and 3 h post-injection. No fluorescence signal from the liver was exhibited during transcutaneous in vivo imaging due to its deep anatomical location. The stronger fluorescence signals of GnRHa-PEG-Rh760 in tumors lasted for up to 72 h post-injection, with a high ratio of tumor-to-background (skeletal muscle) (Figure 2C). Confocal fluorescence microscopy also confirmed the specific binding of GnRHa-PEG-Rh760 in tumors (Figure 2D).

The biodistribution of GnRHa-PEG-Rh760 was evaluated in mice bearing subcutaneous A2780 ovarian cancer. The fluorescence intensities of ex vivo tumors and organs at different time points are shown in Supplementary Figure S2. GnRHa-PEG-Rh760 mainly accumulated in the tumor and liver tissues and was gradually cleared at 96 h post-injection. These data demonstrated the specific accumulation and long retention time of GnRHa-PEG-Rh760 in tumors expressing GnRHR, making it suitable for in vivo imaging.

### 3.3. Specific Accumulation of GnRHa-PEG-Rh760 in Peritoneal Metastases of Ovarian Cancer

**Figure 3:**
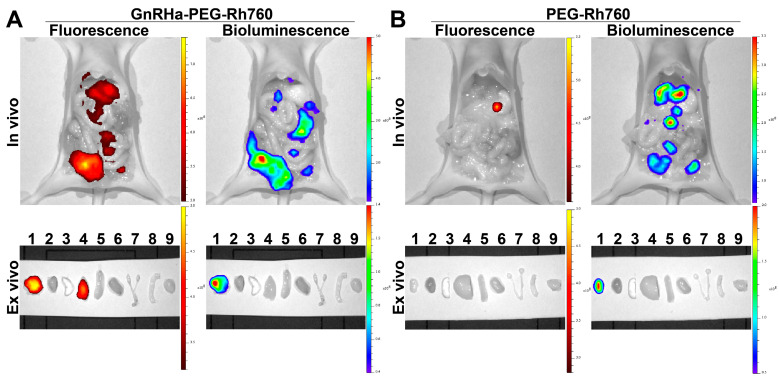
Specific accumulation of GnRHa-PEG-Rh760 in peritoneal metastases of ovarian cancer. Mice bearing peritoneal metastatic A2780-luciferase ovarian cancer were injected intraperitoneally with GnRHa-PEG-Rh760 or PEG-Rh760 into the lower right abdominal region. Representative in vivo and ex vivo NIR fluorescence and bioluminescence images of GnRHa-PEG-Rh760 (**A**) and PEG-Rh760 (**B**). 1, tumor; 2, heart; 3, lung; 4, liver; 5, spleen; 6, kidney; 7, uterus and ovary; 8, intestine; 9, muscle.

Then, we investigated the accumulation of GnRHa-PEG-Rh760 in peritoneal metastatic foci using mice bearing peritoneal metastatic A2780-luciferase ovarian cancer, given that ovarian cancer typically spreads throughout the peritoneal cavity. Similar to the in vivo fluorescence imaging results in the mice bearing subcutaneous xenografts, high fluorescent signals in the peritoneal metastases were observed in the GnRHa-PEG-Rh760 group (Figure 3A), whereas few signals were observed in the PEG-Rh760 control group (Figure 3B). The bioluminescence signals from the luciferase expressed in A2780 cancer cells further verified the NIR fluorescence signals from the accumulated GnRHa-PEG-Rh760 in the tumors. The ex vivo NIR fluorescence and bioluminescence imaging of resected metastasis and major organs validated the targeted accumulation of GnRHa-PEG-Rh760 in peritoneal metastases of ovarian cancer.

### 3.4. Specific Binding and Imaging of GnRHa-PEG-Rh760 for Lymph Node Metastases of Ovarian Cancer

**Figure 4:**
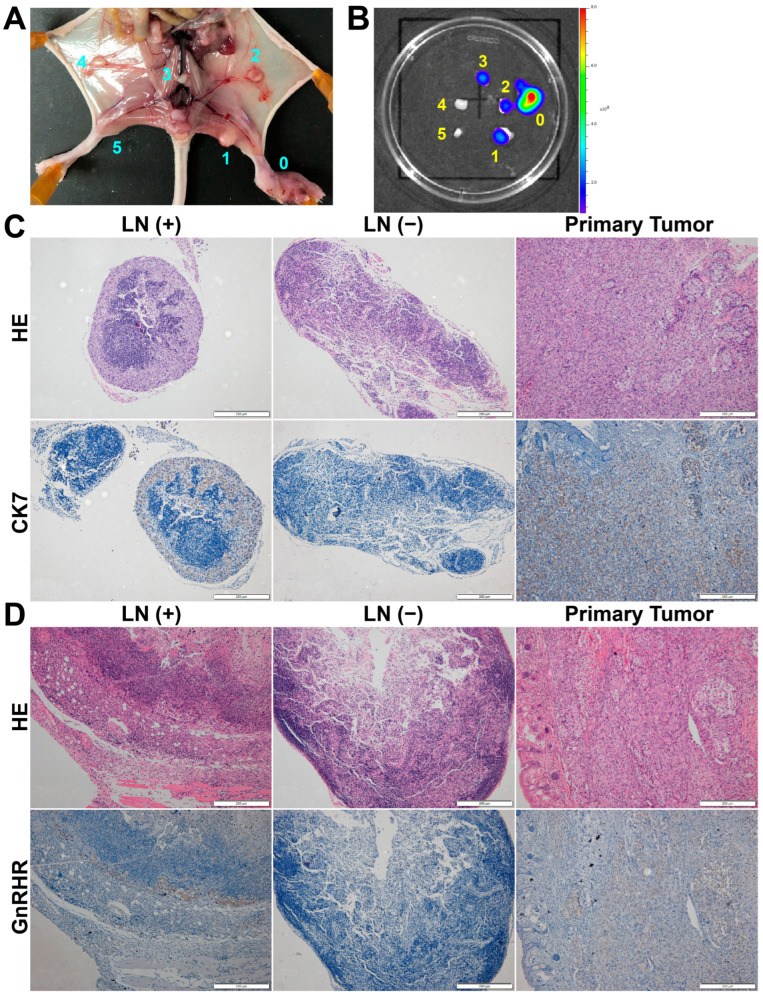
Establishment of a mouse model bearing lymph node metastases of ovarian cancer. The mouse model was established by implanting ES-2-luciferase ovarian cancer cells into the foot pads of BALB/c nude mice. (**A**) White-light image of the gross anatomy of the mice. (**B**) Ex vivo bioluminescence images. 0, primary tumor; 1 and 5, left and right popliteal lymph node; 2 and 4, left and right inguinal lymph node; 3, iliac lymph node. (**C**) H&E and immunohistochemical staining for CK7. (**D**) H&E and immunohistochemical staining for GnRHR. Bar: 200 μm. LN (+), metastatic lymph node; LN (−), normal lymph node.

We further evaluated the binding and imaging of GnRHa-PEG-Rh760 for metastatic lymph nodes using a mouse model with lymph node metastasis of ovarian cancer. As shown in Figure 4A–C, the metastatic lymph nodes exhibited bioluminescence originating from ES-2 cancer cells and showed positive expression of CK7. The pattern of lymph node metastasis was consistent with lymphatic drainage after injecting dye into the mouse footpad [25]. Furthermore, both the primary tumor in the foot pads and the metastatic lymph nodes presented positive GnRHR expression, providing recognition and binding sites for GnRHa-PEG-Rh760 (Figure 4D).

**Figure 5:**
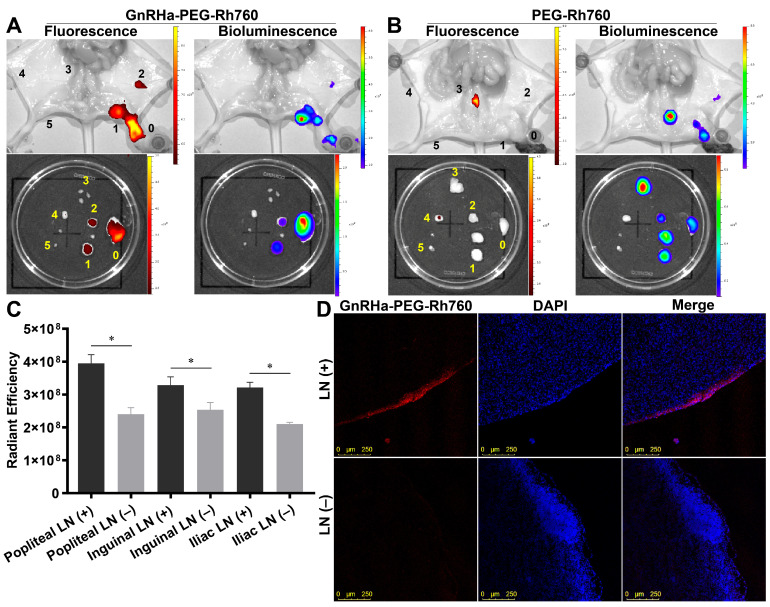
Specific binding and imaging of GnRHa-PEG-Rh760 for lymph node metastases of ovarian cancer. Mice bearing lymph node metastases of ES-2-luciferase ovarian cancer were injected with GnRHa-PEG-Rh760 or PEG-Rh760 via the tail vein. Representative in vivo and ex vivo NIR fluorescence and bioluminescence images of GnRHa-PEG-Rh760 (**A**) and PEG-Rh760 (**B**). 0, primary tumor; 1 and 5, left and right popliteal lymph node; 2 and 4, left and right inguinal lymph node; 3, iliac lymph node. (**C**) Average fluorescence intensities of metastatic and normal lymph nodes post-injection of GnRHa-PEG-Rh760 (n = 3). * *p* < 0.05. (**D**) Representative confocal fluorescence microscopy images of frozen sections from lymph nodes. Bar: 250 μm. LN (+), metastatic lymph node; LN (–), normal lymph node.

As shown in Figure 5A, in the GnRHa-PEG-Rh760 group, the metastatic lymph nodes were well identified and detected by NIR fluorescence, whereas no NIR fluorescence signals were detected in normal lymph nodes, which was consistent with the bioluminescence signals from ES-2-luciferase cancer cells. In contrast, almost no NIR fluorescence signals were detected in either metastatic or normal lymph nodes in the PEG-Rh760 control group (Figure 5B). Furthermore, the average fluorescence intensity of GnRHa-PEG-Rh760 in metastatic lymph nodes was significantly greater than that in normal lymph nodes (Figure 5C). Confocal fluorescence microscopy also confirmed the specific binding of GnRHa-PEG-Rh760 in metastatic lymph nodes (Figure 5D). Collectively, these data demonstrated that GnRHa-PEG-Rh760 was able to specifically bind to and non-invasively image both primary and metastatic tumors as well as lymph nodes in vivo.

### 3.5. In Vivo Safety Evaluation of GnRHa-PEG-Rh760

**Figure 6:**
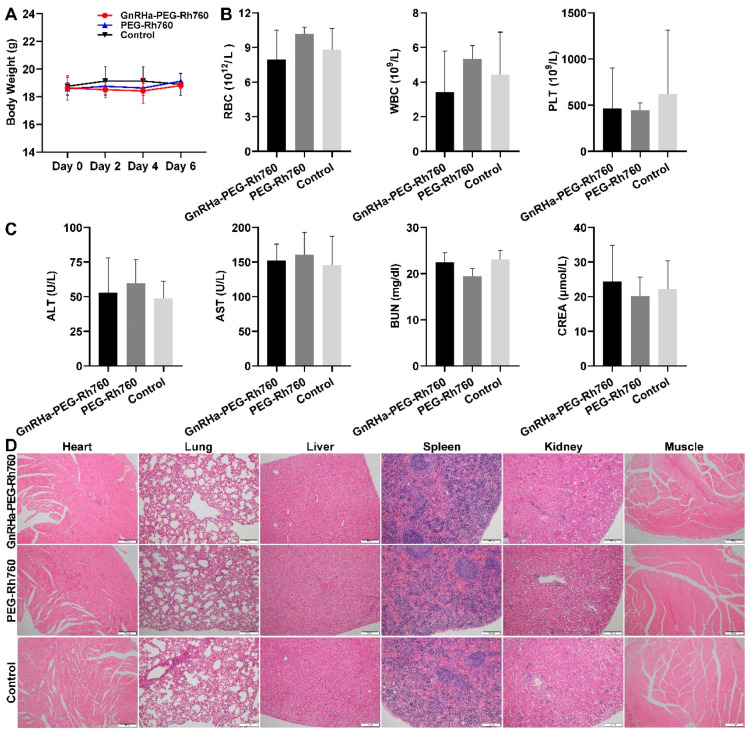
In vivo safety evaluation of GnRHa-PEG-Rh760. The mice were injected with GnRHa-PEG-Rh760, PEG-Rh760 or vehicle control via the tail vein (n = 3). Hematologic and histopathological analyses of the organs were performed after 6 days of treatment. (**A**) Body weights of the mice. (**B**) Routine blood tests, including red blood cell (RBC), white blood cell (WBC) and platelet (PLT) counts. (**C**) Blood biochemistry tests, including alanine transaminase (ALT), aspartate transaminase (AST), blood urea nitrogen (BUN), and creatinine (CREA) levels. (**D**) H&E staining of organs, including the heart, lung, liver, spleen, kidney and muscle. Bar: 200 μm.

To evaluate the safety and biocompatibility of GnRHa-PEG-Rh760, the mice were intravenously injected with GnRHa-PEG-Rh760, PEG-Rh760 or vehicle control and were sacrificed after 6 days. No significant body weight loss was noted following the injection of GnRHa-PEG-Rh760 or PEG-Rh760 compared with the control group (Figure 6A). Hematologic analysis revealed no significant changes in RBC, WBC, PLT, ALT, AST, BUN, or CREA levels (Figure 6B,C). No notable tissue injury was observed in the heart, lung, liver, spleen, kidney, or muscle in either group (Figure 6D). These results indicated that GnRHa-PEG-Rh760 was safe and biocompatible for in vivo imaging.

## 4. Discussion

Real-time and accurate intraoperative identification and resection of metastatic tumors and lymph nodes are crucial for optimizing surgical outcomes in ovarian cancer patients. In this study, we synthesized and characterized a GnRHR-targeted NIR fluorescent probe, GnRHa-PEG-Rh760, and demonstrated its specific binding and imaging capabilities for primary tumors, peritoneal metastases, and lymph node metastases in ovarian cancer.

Intraoperative fluorescence imaging offers real-time visualization of lesions, facilitating precise tumor resection and preventing the resection of normal tissues [26,27,28]. The accurate identification of tumor lesions relies on the targeted capability of fluorescent tracers [29,30]. The targeting carriers, such as peptides, antibodies, and small molecules, conjugated to fluorescent dyes bind with specific cellular surface targets that are strongly expressed in cancer tissues relative to surrounding noncancer tissues [9,31]. Specific targets and high-affinity ligands are still being explored. GnRHR is overexpressed in hormone-related cancers (e.g., 80% of ovarian cancer, 85% of endometrial cancer, 50% of breast cancer, and 86% of prostate cancer), as well as hormone-unrelated cancers (e.g., melanoma, glioblastoma, lung cancer and pancreatic cancer) [32,33,34]. Moreover, the expression of GnRHR in metastatic lymph nodes is equal to or greater than that in primary tumors [35]. Owing to the selective expression of GnRHR in cancers but not in most normal tissues, GnRHR could be an effective theranostic target to guide the delivery of anticancer therapeutics and diagnostic agents into cancer cells [36]. Our previous studies have also confirmed the overexpression of GnRHR in the majority of ovarian cancer samples [22]. Thus, in this study, GnRHR was selected as the tumor target, and the GnRH derivative was used as the targeting carrier of the fluorescent probe.

GnRH, a conserved decapeptide sequence among mammals, plays a pivotal role in regulating reproductive physiology. Unlike endogenous GnRH, GnRH analogs or derivatives exhibit prolonged receptor binding and enhanced resistance to enzyme degradation, with widespread clinical applications [37]. In preclinical studies, GnRH derivatives have been used as targeting carriers in peptide-based drug delivery platforms, enhancing the selective delivery of payloads into cancer cells via GnRHR-mediated endocytosis [34,38,39,40,41]. By conjugating the GnRHa peptide to the Rh760 fluorophore via a PEG linker, we ensured both tumor-targeting capability, favorable biodistribution profile and biocompatibility. The small molecule NIR fluorophore Rh760 has shown stable in vivo imaging capacities in our previous studies [23,24]. The spectra of GnRHa-PEG-Rh760 matched those of the non-targeted PEG-Rh760, confirming that the conjugation of the peptide did not compromise the optical properties of the fluorophore. Compared with the non-targeted control probe, GnRHa-PEG-Rh760 exhibited sustained retention in GnRHR-positive tumors, as evidenced by the prolonged NIR fluorescence signal in the mice, allowing for a flexible time window for operation. Our study reinforced the great potential of the GnRHR-targeting strategy in molecular imaging.

The biodistribution profile of GnRHa-PEG-Rh760 was similar to that of other PEGylated agents, with predominant accumulation in the tumor and liver, followed by the kidneys [42,43]. The long-term toxicity resulting from high accumulation in the liver and kidneys warrants careful consideration prior to clinical application. In addition, the observed weak NIR fluorescence in normal ovarian tissues aligns with physiological GnRHR expression in reproductive organs, indicating the probe’s specificity for GnRHR-positive tissues. Although our safety assessment showed no hematologic or organ toxicity, likely attributable to the low dosage of the imaging probe and its non-therapeutic purpose, this aspect demands careful evaluation for clinical application, particularly in gynecologic tumors scheduled for fertility-sparing surgery or tumors unrelated to the reproductive system.

The incidence of lymph node metastases is 14.2% (range 6.1%–29.6%) in patients with clinical stages I and II ovarian cancer [44] and is greater than 50% in patients with advanced-stage ovarian cancer [15,17,45]. Systematic lymphadenectomy does not improve survival but instead leads to more postoperative complications, highlighting the necessity of preoperative and intraoperative assessment of lymph nodes to indicate whether lymphadenectomy should be performed [17,18]. Intraoperative recognition and selective resection of metastatic lymph nodes could improve the clinical outcome of patients with ovarian cancer. However, intraoperative palpation of lymph nodes, even by experienced gynecologic oncologists, has low sensitivity and positive predictive value [46]. Here, GnRHa-PEG-Rh760 illuminated metastatic lymph nodes while sparing normal lymph nodes, as evidenced by the precise co-localization of NIR signals with bioluminescence signals from cancer cells. In addition to the sentinel metastatic lymph node (popliteal), distant metastatic lymph nodes (inguinal and iliac) could also be identified. This can be attributed to the fact that GnRHa-PEG-Rh760 specifically targets the GnRHR, which is overexpressed in metastatic lymph nodes. Binding of the GnRHa-conjugated probe to its receptors allows for selective accumulation in GnRHR-positive tumors [47]. This receptor-mediated accumulation provides the basis for real-time surgical navigation, enabling visualization of metastatic foci and selective resection. However, it should be noted that the binding specificity of the probes may vary depending on GnRHR expression heterogeneity.

In this study, the administration route of the probe was tailored to the different mouse models in order to explore its potential in various clinical scenarios. For mice bearing subcutaneous xenografts and lymph node metastasis, systemic administration via tail vein was used to assess the probe’s tumor-targeting capability across the body. For mice bearing peritoneal metastasis, intraperitoneal injection in the lower right abdominal region was chosen to allow direct binding of the probe to peritoneal tumors. Although in vivo imaging demonstrated co-localization of probe-derived fluorescence and tumor-specific bioluminescence in key peritoneal metastatic sites, in vivo signal discrepancies were observed in several regions. The absence of fluorescence in upper abdominal tumors may be attributed to insufficient diffusion and binding of the probe following intraperitoneal injection, particularly in large metastatic tumors. Additionally, the regular rodent diet contains alfalfa, a source of chlorophyll which can cause strong gastrointestinal autofluorescence, leading to non-specific signals [48]. To minimize autofluorescence during abdominal fluorescence imaging in mice, it is advisable to use a low-fluorescence diet and implement prolonged fasting.

While this study demonstrates the potential of GnRHa-PEG-Rh760 for in vivo imaging, several limitations should be acknowledged. The absence of luciferase labeling in the subcutaneous xenografts, as well as the absence of H&E staining, limits direct correlation between the fluorescence signals of the probes and tumor regions. Multiple labeled co-localization of the probes and tumors could be employed in future studies. Although statistically significant differences were found and the probe’s tumor-targeting imaging capability was validated across different mouse models, the relatively small sample size might compromise statistical power. Additionally, the fluorescence quantum yield of Rh760 within the probe formulation was not quantified, limiting precise brightness comparisons with other fluorescent probes. Although acute toxicity studies showed no organ damage, long-term biosafety assessments are essential to exclude delayed toxicity. The systemic pharmacokinetic profile of the probes requires further evaluation prior to clinical translation.

## 5. Conclusions

In summary, this study successfully developed a GnRHR-targeted NIR fluorescent probe, which illuminated primary tumors, peritoneal metastases and lymph node metastases of ovarian cancer with high specificity in preclinical models. This probe could be a promising tool for intraoperative fluorescence imaging guidance in precision cancer surgery. Future research could develop multifunctional theranostic probes that integrate tumor-targeted imaging and therapeutic functions, achieving the integration of diagnosis and treatment. Furthermore, employing artificial intelligence algorithms to automatically analyze fluorescent images could provide valuable support for real-time lesions identification and intraoperative decision-making.

## Data Availability

The original contributions presented in this study are included in the article/supplementary material. Further inquiries can be directed to the corresponding authors.

## Supplementary Materials

The following supporting information can be downloaded at: https://www.mdpi.com/article/10.3390/biom15060868/s1, Figure S1: Mass spectra of GnRHa-PEG-Rh760 (A) and PEG-Rh760 (B); Figure S2: Biodistribution of GnRHa-PEG-Rh760 in mice bearing subcutaneous A2780 ovarian tumors.
