# Supplementary material for: A Near-Infrared Fluorescent Probe for Specific Imaging of Lymph Node Metastases in Ovarian Cancer via Active Targeting of the Gonadotropin-Releasing Hormone Receptor
Source: Biomolecules. 2025 Jun 14;15(6):868. doi: 10.3390/biom15060868 (PMC12190659; doi:10.3390/biom15060868)
Supplement: Supplementary file 1 [file biomolecules-15-00868-s001.zip › biomolecules-3653482-supplementary.pdf]

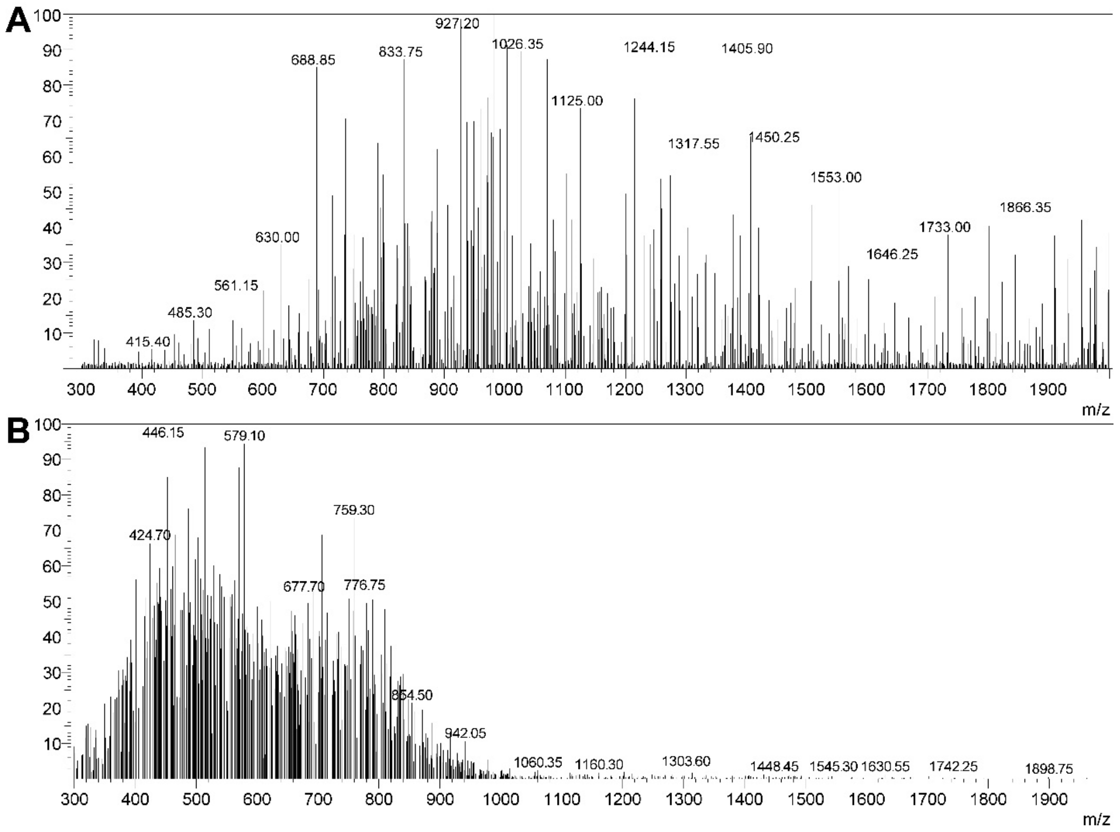

Figure S1 Mass spectra of GnRHa-PEG-Rh760 (A) and PEG-Rh760 (B).

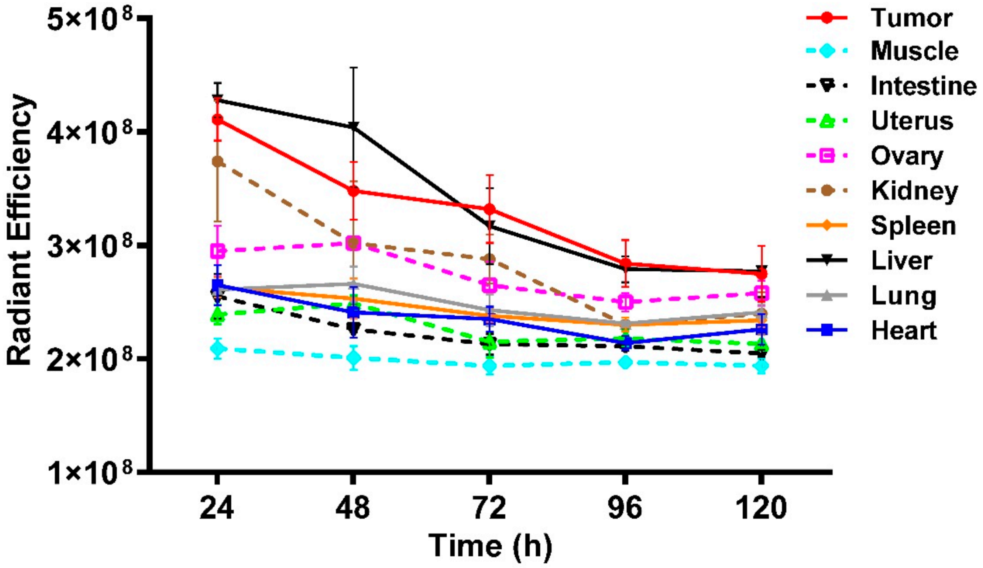

Figure S2 Biodistribution of GnRHa-PEG-Rh760 in mice bearing subcutaneous A2780 ovarian tumors. The mice were injected with GnRHa-PEG-Rh760 via the tail vein and monitored for 120 h. The fluorescence intensities of the *ex vivo* tumors and organs were measured (n=3).
